# Thermal stress: an untapped potential in rehabilitation? An opinion paper on Finnish sauna and whole-body cryostimulation

**DOI:** 10.3389/fresc.2026.1720021

**Published:** 2026-05-29

**Authors:** Angelo Alito, Federica Verme, Lara Costa, Calogero Malfitano, Anamaria Gherle, Sara Laxe, Jacopo Maria Fontana, Paolo Capodaglio

**Affiliations:** 1 Department of Biomedical, Dental Sciences and Morphological and Functional Images, University of Messina, Messina, Italy; 2 Laboratory of Biomechanics, Rehabilitation and Ergonomics, Istituto Auxologico Italiano, IRCCS, S. Giuseppe Hospital, Piancavallo, Italy; 3 Department of Clinical and Experimental Medicine, University of Messina, Messina, Italy; 4 Department of Biomedical Sciences for Health, University of Milan, Milan, Italy; 5 Azienda di Servizi Alla Persona Istituti Milanesi Martinitt e Stelline e Pio Albergo Trivulzio, Milan, Italy; 6 Psychoneuro Sciences and Rehabilitation Department, Faculty of Medicine & Pharmacy, University of Oradea, Oradea, Romania; 7 Physical and Medicine Department, Hospital Clinic Barcelona, Barcelona, Spain; 8 Department of Medicine, Rheumatology Unit, Center for Molecular Medicine, Karolinska Institute, Stockholm, Sweden; 9 Department of Biomedical, Surgical and Dental Sciences, University of Milano, Milan, Italy

**Keywords:** cold therapy, cryotherapy, Finnish sauna, hot temperature, rehabilitation, sauna, thermal stress, whole-body cryostimulation

## 1 Introduction

Thermal therapies, from ancient baths to modern cryochambers, have long been used for their physiological effects. Historically, heat was employed by Egyptians and Romans for pain and fever, while cold applications have transitioned from traditional medicine to modern cryotherapy for their analgesic and anti-inflammatory properties (1–4). Contrast therapy, alternating heat and cold, is thought to induce a “vascular pump” effect, reducing oedema (5–7).

Despite centuries of use, evidence remains fragmented. Recent reviews highlight modest effects—such as improved blood flow and reduced soreness—but emphasize a lack of standardized protocols and methodological quality (5, 8). This uncertainty persists while patients often prefer “passive” treatments, especially in early rehabilitation stages where kinesiophobia, pain, and low motivation hinder active exercise (9–13). Adherence to exercise programs often falls below 70% due to discomfort or perceived lack of progress (14).

Recently, Finnish sauna (FS) and whole-body cryostimulation (WBC) have gained attention. These passive modalities offer immediate sensory relief and may alleviate movement-related anxiety (14, 15). FS (80–100°C) is associated with improved cardiovascular function and autonomic regulation (16), while WBC (–110 to −140°C) modulates pro-inflammatory cytokines and sympathetic tone (17–19). A crucial mechanism likely involves the vagus nerve, which regulates inflammation and stress responses. Both heat and cold can influence vagal tone, promoting systemic homeostasis (20–23). Although heat triggers sympathetic activation, repeated exposure (sauna) induces a parasympathetic shift, reflected in increased heart rate variability (HRV) (24, 25).

This opinion paper aims to explore the current scientific evidence regarding the use of FS and WBC in clinical rehabilitation. It examines their physiological mechanisms, therapeutic applications, and the feasibility of integrating them into multidisciplinary programs.

## 2 Systemic heat-based treatment (FS)

FS involves brief exposure to dry air (80–100°C) with 10%–20% humidity. Sessions typically last 5–20 min, increasing heart rate to 120–150 bpm, mimicking moderate aerobic stress (26–28).

### 2.1 Physiological evidence

Whole-body heat therapy triggers adaptations primarily through several mechanisms such as:

Vascular Shear Stress: Enhances endothelial nitric oxide synthase (eNOS) activity, increasing nitric oxide (NO) bioavailability, which improves arterial elasticity and peripheral circulation (29, 30).

Heat Shock Proteins (HSPs): Increased HSP70 and HSP90 levels protect protein integrity, mitigate apoptosis, and shield cells from oxidative stress (31–33).

Anti-inflammatory Effects: Reduction in pro-inflammatory markers and oxidative stress contributes to metabolic resilience (34, 35).

Autonomic Regulation: Repeated thermal exposure induces a enhances autonomic flexibility, improving the body's ability to dynamically balance sympathetic activation with parasympathetic recovery, characterized by increased HRV and a reduction in resting sympathetic tone (25).

Nociception: Heat activates TRPV1 channels on sensory neurons, modulating C-fiber activity and stimulating analgesic responses via central mechanisms (36, 37).

### 2.2 Clinical effects of FS bathing

Habitual sauna use is linked to reduced risks of hypertension, cardiovascular disease, and neurodegenerative disorders (31, 38, 39). In rehabilitation, FS alleviates musculoskeletal pain, osteoarthritis, and chronic headaches (16, 36). It modulates autonomic balance, reducing sympathetic tone while increasing parasympathetic activity during recovery (16, 40). Notably, the levels of norepinephrine reached during sauna exposure are comparable to those observed after maximal exercise, indicating a potent autonomic stimulus (36). Hormonal responses include transient increases in norepinephrine, cortisol, and growth hormone, which may confer adaptive benefits (34, 41).

Preliminary evidence suggests that heat therapy may influence circulating biomarkers of glucose and lipid metabolism, insulin resistance and cardiac injury (e.g., troponin T); however, further studies are required to establish these associations definitively (42, 43).

### 2.3 Safety and contraindications

FS is generally safe for stable coronary artery disease or compensated heart failure (44, 45). However, it is contraindicated in unstable angina, recent myocardial infarction, severe aortic stenosis, or uncontrolled hypertension (25, 46). Combining sauna with cold plunges can trigger dangerous autonomic surges in vulnerable individuals (34, 45). Alcohol consumption is strictly discouraged due to the risk of hypotension and impaired thermoregulation (25). Furthermore, FS is contraindicated for individuals prone to syncope or orthostatic intolerance, as the significant peripheral vasodilation may exacerbate hemodynamic instability (25). Caution is also required for thermosensitive Multiple Sclerosis (MS) patients, as elevated internal temperatures can lead to a transient increase in neurological symptoms (46, 47).

### 2.4 Knowledge gaps

Despite documented benefits, the clinical application of FS in rehabilitation remains limited. Most evidence is observational or based on general cardiovascular cohorts, leaving a void in standardized protocols for multidisciplinary frameworks (16, 29). Specifically, a lack of RCTs targeting functional recovery in stroke, pulmonary disease, or complex musculoskeletal populations prevents the translation of sauna-induced physiological gains into motor or cognitive outcomes. Furthermore, while safe for stable cardiac patients, the absence of data on physical frailty or severe autonomic dysregulation hinders its broader utility. Future research must define precise therapeutic “dosing”—temperature, duration, and frequency—to responsibly integrate passive heat into mainstream rehabilitative care.

### 2.5 Potential applications

The systemic effects of FS have a wide range of applications in rehabilitative medicine. In cardiovascular rehabilitation, for example, heat exposure can be used alongside treatment for stable coronary artery disease or heart failure to improve endothelial function and reduce vascular resistance (29, 34). For chronic pain conditions such as fibromyalgia, osteoarthritis and rheumatoid arthritis, FS has been shown to be effective in reducing joint stiffness and elevating pain thresholds through nociceptor modulation (26, 36, 42).

In respiratory and metabolic care, preliminary evidence suggests improved lung function in obstructive pulmonary diseases and favorable glucose and lipid modulation in metabolic syndrome (42, 43). Beyond the physical benefits, the psychological impact, including reduced anxiety and improved sleep, supports neurorehabilitation by enhancing patient motivation for active exercise (41). While the use of FS in post-stroke recovery and in the treatment of neurodegenerative diseases shows promise due to improved autonomic balance, further validation through rigorous clinical trials is required in these areas.

## 3 Systemic cold-based treatments

WBC involves brief exposure to −110°C to −160°C air for 1–3 min, triggering neuroendocrine, immune, and circulatory adaptations (48–50). It has recently emerged as a promising non-pharmacological intervention in the rehabilitation field to modulate chronic pain, reduce neuroinflammation, and serve as a “priming” tool to facilitate the transition to active therapeutic exercise (51).

### 3.1 Emerging physiological evidence

Extreme cold triggers systemic adaptations through several physiological pathways, such as:

Neuroendocrine Activation: stimulation of the hypothalamic-pituitary-adrenal (HPA) axis and a simultaneous stimulation of the sympathetic-adrenal-medullar*y* axis to release adrenaline and noradrenaline, enhancing arousal (52).

Vascular Modulation: Initial catecholamine-induced vasoconstriction followed by reactive vasodilation, which improves tissue perfusion and microvascular reactivity (53).

Immune Response: Downregulation of pro-inflammatory cytokines (such as TNF-α, IL-6, and IL-1β) and upregulation of the anti-inflammatory cytokine IL-10 (54, 55).

Nociceptive Signaling: Activation of TRPM8 ion channels and release of β-endorphins, which suppress pain signaling and elevate pain thresholds (56, 57).

Emerging evidence also suggests neuroprotective potential through cold-shock proteins (e.g., RBM3) and brain-derived neurotrophic factor (BDNF) modulation (50, 58).

### 3.2 Clinical effects

WBC serves as a multi-target adjunctive therapy in rehabilitation. It supports metabolic health by activating brown adipose tissue (BAT) and enhancing thermogenic lipolysis, aiding in weight management and insulin sensitivity (59–61). In neurological and pain syndromes, it reduces fatigue, improves sleep quality, and alleviates symptoms of Multiple Sclerosis (MS), fibromyalgia, and central sensitization (47, 62, 63). In post-COVID-19 rehabilitation, especially for patients with obesity, WBC facilitates engagement in exercise by reducing musculoskeletal discomfort and systemic inflammation (53). WBC has also been shown to exert anti-inflammatory effects, primarily through a shift in cytokine profiles such as TNF-α, IL-6, and IL-1β, while promoting an increase in IL-10, a key anti-inflammatory cytokine (64, 65).

### 3.3 Adverse events and contraindications

WBC is generally safe in controlled settings, though transient side effects like shivering, mild headache, and skin redness can occur (66). Rare but significant reactions include blood pressure spikes, transient arrhythmias, and vasospastic responses, particularly in patients with compromised autonomic function (67). Adverse events like cold urticaria or frostbite are typically associated with inadequate medical screening or safety protocol violations (66, 68, 69).

Current consensus distinguishes between absolute, relative, and situational contraindications to ensure safe clinical application (70). Absolute contraindications include unstable cardiac conditions (recent MI, severe aortic stenosis), cold-induced disorders (Raynaud's II/III, cryoglobulinemia), acute DVT, and uncontrolled epilepsy. Relative or situational contraindications include controlled arrhythmia, mild autonomic dysfunction, fever, or recent surgery. Proper risk stratification, comparable to ergometric testing, is essential for the safe integration of WBC into multidisciplinary rehabilitation.

With proper risk stratification, WBC can be administered safely in sports and clinical rehabilitation settings.

### 3.4 Knowledge gaps

Despite clinical promise, WBC lacks established consensus on optimal parameters (temperature, duration, and frequency), hindering the formulation of standardized protocols (70). Many mechanisms remain inadequately elucidated or extrapolated from small, uncontrolled cohorts (18, 51, 59). There is a scarcity of direct clinical investigations into long-term metabolic effects in obesity and a lack of robust randomized controlled trials (RCTs) with validated functional outcomes for chronic pain. Future research must prioritize uniform protocols, mechanistic clarity, and longitudinal cost-effectiveness studies.

### 3.5 Potential application

WBC is increasingly utilized as an adjunctive therapy for chronic conditions due to its systemic effects on inflammation, metabolism, and neuroendocrine function. In neurological rehabilitation, WBC has shown significant benefits for patients with MS, specifically improving fatigue, sleep quality, mood, and functional performance (47, 71). Similarly, in chronic pain syndromes like fibromyalgia, WBC demonstrates substantial reductions in pain severity, anxiety, and disease impact, with benefits sustained over time (55, 62, 66, 72).

In post-COVID-19 rehabilitation, particularly for individuals with obesity, WBC integrated into multidisciplinary protocols has resulted in meaningful reductions in body mass, systemic inflammation, and perceived fatigue (53). Its applications also extend to neuropathic and musculoskeletal pain, including phantom limb pain and kinesiophobia in chronic shoulder disorders, where it enhances patient engagement by providing immediate symptomatic relief (58, 73, 74). While preliminary evidence in Parkinson's disease (75), spasticity (76), and rheumatic disorders (51, 77) is promising, robust clinical trials are necessary to define optimal protocols and long-term outcomes.

## 4 Discussion

**Figure 1:**
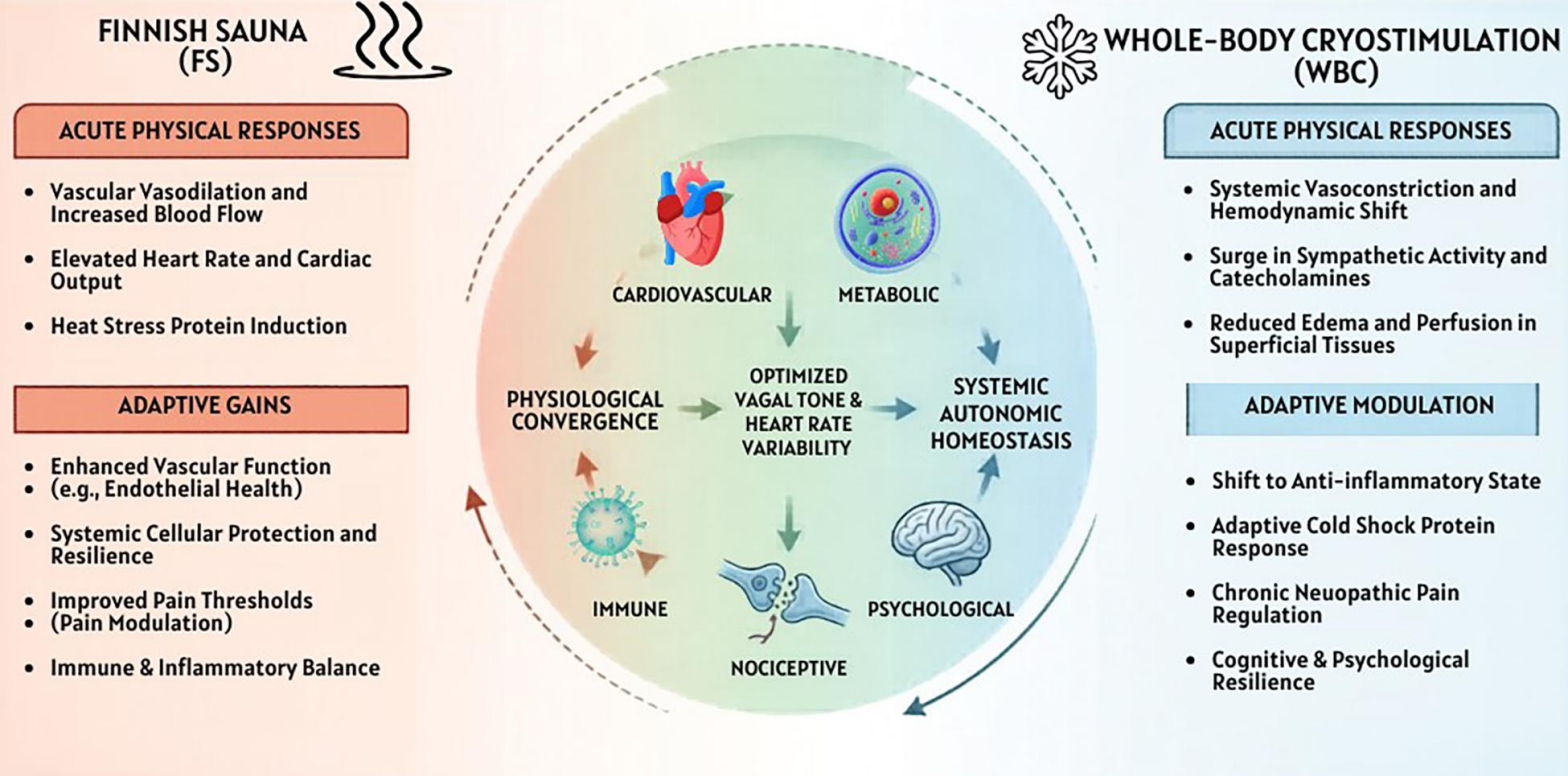
This picture illustrates the use of FS and WBC as physiological “priming” tools. The lateral panels distinguish between acute responses and adaptive gains for both the heat and cold modalities. These pathways converge in the central zone, optimizing vagal tone and HRV and promoting systemic autonomic homeostasis. This provides symptomatic relief and facilitates the clinical transition to active exercise protocols.

Integrating systemic thermal therapies such as FS and WBC into rehabilitation represents a paradigm shift, moving away from purely active models and towards “priming” approaches that are consistent with interventions such as nature exposure and mind-body practices (78–80). These modalities act as physiological boosters, which are particularly beneficial during the early stages of recovery when kinesiophobia, pain and fatigue can hinder exercise. By modulating the autonomic nervous system, specifically via the vagus nerve, thermal stress can improve systemic homeostasis and psychological readiness (25, 46). This convergence towards optimized autonomic flexibility and HRV creates a “priming zone” (see Figure 1). This homeostatic window optimizes the physiological response, reducing kinesiophobia and allowing the transition to active exercise protocols to be facilitated.

However, translating these biological signals into clinical practice remains challenging. One critical issue is the “dose-response” relationship: the ideal temperature, duration and frequency remain undefined for most pathological cohorts. Furthermore, while preclinical data on heat shock proteins (HSPs) and cold-shock proteins (RBM3) suggest neuroprotective mechanisms, their role in human recovery remains speculative (81). The absence of validated biomarkers further complicates efforts to personalize treatment.

Another significant gap lies in the interaction between thermal stress and other rehabilitation components. It is currently unclear whether thermal therapies act synergistically with specific exercise protocols, or whether concurrent medications alter the expected cardiovascular and immune responses. Moreover, most research focuses on the acute effects; the long-term sustainability of the benefits, and whether a “maintenance dose” is required, remain largely unexplored.

### 4.1 Limits

This paper is limited by the heterogeneous nature of current literature and the lack of systematic appraisal, which may introduce selection bias. The reliance on pilot studies, case reports, and surrogate markers limits firm conclusions about efficacy and safety. Additionally, the lack of robust comparative data between heat and cold modalities makes it difficult to define specific indications for each. Mechanisms like BAT activation or neuroimmune modulation require further study in human clinical settings. Until high-quality RCTs are available, these promising applications should be interpreted with caution.

## 5 Conclusion

Systemic heat- and cold-based therapies, such as FS and WBC, are emerging as promising additional strategies in the field of rehabilitation medicine. Their ability to trigger diverse systemic responses, including cardiovascular, neuromuscular, metabolic, autonomic and immunological responses, makes them valuable tools for addressing chronic pain, fatigue and neuroinflammation. Although these interventions are traditionally viewed as “passive”, they can act as a rehabilitation booster, particularly during the early stages of recovery when pain and deconditioning often impede progress.

Furthermore, high levels of patient satisfaction and immediate symptomatic relief can help to overcome common barriers such as low motivation and poor treatment adherence. However, despite the encouraging preliminary findings, their integration into structured protocols is limited by a lack of standardized dosing and long-term evidence. Robust clinical trials are essential to establish therapeutic guidelines and clarify interactions with other interventions. Until then, these modalities should be considered valuable yet experimental components of multidisciplinary care and only be prescribed following rigorous medical screening and individualized clinical reasoning.
